# A cross-sectional study of predictive factors of health literacy among rheumatoid arthritis patients in China

**DOI:** 10.3389/fpsyg.2024.1390442

**Published:** 2024-06-27

**Authors:** Ting Liu, Weifen Meng, Wenlong Wang, Guomin Sun, Xi Chen, Yan Lu, Weiping Qin, Yan Wang, Lu Zhang, SuHua Zheng

**Affiliations:** ^1^Department of Rheumatology, The Affiliated Changzhou No.2 People’s Hospital of Nanjing Medical University, Changzhou, Jiangsu, China; ^2^Department of Rheumatology, The First People's Hospital of Wenling, Wenling, Zhejiang, China; ^3^Department of Rheumatology, Affiliated Hospital of Nanjing University of Chinese Medicine, Jiangsu Province Hospital of Chinese Medicine, Nanjing, Jiangsu, China; ^4^Department of Rheumatology, Changzhou Community Health Service Center, Changzhou, Jiangsu, China

**Keywords:** rheumatoid arthritis, health literacy, self-efficacy, medication adherence, disability index

## Abstract

**Objective:**

To investigate the factors that influence health literacy (HL) among Chinese patients with rheumatoid arthritis (RA) and furnish theoretical underpinnings for the development of intervention strategies aimed at enhancing patients’ quality of life.

**Methods:**

From May 2022 to December 2022, a comprehensive survey was conducted among both outpatients and inpatients diagnosed with (RA) in a tertiary hospital in China. The survey utilized various instruments, including a general information questionnaire, a chronic disease patient health literacy scale, the Health Assessment Questionnaire-Disability Index (HAQ-DI), the Chinese-translated Rheumatoid Arthritis Self-Efficacy Scale, the Chinese-translated Rheumatoid Arthritis Stigma Scale, and the Chinese-translated Compliance Questionnaire for Rheumatology Treatments.

**Results:**

The average scores of HL, self-efficacy, medication adherence, and disability index were 83.54 ± 17.43, 84.91 ± 14.37, 70.16 ± 11.24, and 0.26 ± 0.44, respectively. HL in Chinese RA patients was negatively correlated with age, erythrocyte sedimentation rate (ESR), number of tender joints, number of swollen joints, and disease activity, while positively correlated with self-efficacy and medication adherence. Age, disease activity, disability index, self-efficacy, and medication adherence are predictive factors of HL, and a predictive model has been initially constructed.

**Conclusion:**

In the management of RA, healthcare professionals should develop and implement effective intervention measures by focusing on improving medication adherence, enhancing patients’ self-efficacy, improving patients’ physical function, and reducing disease activity. This will help enhance the health literacy and promote clinical outcomes in RA patients.

## Introduction

Rheumatoid arthritis (RA) is a chronic autoimmune disease characterized by inflammatory arthritis. In China, the prevalence of RA ranges from 0.35 to 0.45%, with an estimated total RA patient count of approximately 5 million, exhibiting a significant male-to-female ratio of 1:4 (Yu et al., 2023). Extensive research has revealed that joint diseases constitute one of the primary sources of physical disability, with RA occupying the topmost position in terms of disability rates (Bai et al., 2020). This high disability rate is attributed to the unclear etiology of RA and the absence of effective curative measures (Brown et al., 2024). Moreover, the scarcity of preventive research and delayed interventions serve as significant factors that contribute to the unfavorable prognosis of RA (Abbasi, 2017).

Health literacy (HL) is a cognitive and social skill that determines an individual’s motivation and ability to access, understand, and apply information to promote and maintain health (Nutbeam, 2008). Research has shown that HL is a crucial factor in health outcomes (Stormacq et al., 2020). Low HL among RA patients can lead to poor medication adherence and an increased risk of misinterpretation of medical advice (Nakayama et al., 2015). HL is not merely a lack of knowledge and skills; it also involves making correct health decisions through self-awareness (Zhuang et al., 2023). Studies have found that the proportion of HL in RA patients ranges from 7 to 42% (Loke et al., 2012). Many factors contribute to low HL in RA patients, including low education levels, a lack of reading habits, as well as race, income, and age-related factors (Martin et al., 2013). A study conducted in a nursing outpatient clinic in Germany revealed a significant association between high HL and low disease activity as well as high quality of life across various dimensions (Hirsh et al., 2019). A cross-sectional study of 110 RA patients found that low HL was associated with joint dysfunction (Caplan et al., 2014). A study in Ontario, Canada, showed that poor HL can lead to poor medication adherence (Gong et al., 2015).

In the early stages, most domestic and international scholars investigated the influencing factors of HL primarily from demographic characteristics. As cross-sectional studies have progressed, social and psychological factors have become hot topics of discussion (Stoll et al., 2024). However, correlation research remains comparatively scarce, and comprehensive, large-scale cross-sectional studies encompassing multiple factors are still pending. The consequence of low HL is suboptimal clinical outcomes, which ultimately impedes the progression toward high-quality healthcare. Therefore, the focus of research has shifted to how to identify the influencing factors of low HL in RA patients from multiple dimensions and perspectives. In terms of psychological factors, a higher level of HL has been linked to patient confidence (Jang et al., 2024). Hence, this study includes the self-efficacy of RA patients in a cross-sectional study on HL. Additionally, patients’ symptoms, disease activity, physical function, and self-management capabilities have also become areas of our focus. Meanwhile, as the commonly used functional HL scales have not undergone reliability and validity testing in the Chinese RA population, this study employs a chronic disease HL scale that is suitable for the Chinese population. The aim of this study is to explore factors significantly associated with HL in RA patients, reveal potential predictors of HL in Chinese RA patients, and initially develop a predictive model. This will provide theoretical support for medical professionals to accurately identify RA patients with low HL levels, develop targeted intervention measures, and improve health outcomes.

## Methods

### Demographic survey and questionnaire

A convenience sampling method was adopted to recruit RA patients from the outpatient and inpatient departments of the rheumatology and immunology division in Changzhou, China, from May 2022 to December 2022. Inclusion criteria were: ages ranging from 18 to 79; diagnosed with RA according to the 1987 American College of Rheumatology (ACR) classification criteria; normal cognitive ability and clear expression skills; disease duration greater than 6 months. Exclusion criteria were: severe personal or family changes affecting the patient’s emotional well-being during the survey period; presence of severe diseases (such as heart failure, renal insufficiency, respiratory failure, severe infection, etc.) and extra-articular manifestations of RA; and the presence of other autoimmune diseases. The survey was conducted by a nurse from the Chronic Rheumatic Disease Management Center. After obtaining the patients’ informed consent, all questionnaires were distributed to the patients, and uniform instructions were provided for any issues encountered during completion. The questionnaires were collected on the spot, and strict confidentiality measures were implemented to protect the participants’ privacy. A total of 172 participants were surveyed in this study, and 172 valid questionnaires were recovered, with a 100% response rate. This study was approved by the Ethics Committee of our hospital with the approval number [2021] KY025-01.

### Chronic disease HL Management Scale

The HL Management Scale (HeLMS), which was developed by Australian scholars, was translated and revised by Sun (2012) in China. The scale comprises 24 items and is divided into 4 dimensions. The total score is 120 points, and a higher score indicates a higher level of HL. Patients scoring 80% or above are defined as having high HL. The Cronbach’s alpha coefficient of this scale ranges from 0.857 to 0.947, indicating good internal consistency of the scale.

### Chinese Compliance Questionnaire for Rheumatology (CCQR)

This scale was translated into Chinese by Zhu et al. (2013). It comprises 19 items, utilizing a 4-point scoring method. The CCQR score is calculated using the formula: (sum of scores for each item-19) /0.57. A score of 80 or above indicates good compliance. The intra-class correlation coefficient for the CCQR score is 0.994 (95% confidence interval: 0.990, 0.997).

### Chinese version of Rheumatoid Arthritis Self-Efficacy Scale (C-RASE)

This scale was adapted into Chinese by Chinese scholars Li et al. (2019). It consists of 25 items, with scores ranging from 1 to 5, and a total score ranging from 25 to 125. A higher score indicates a stronger sense of self-efficacy. The Cronbach’s α coefficient for this scale is 0.751.

### Chinese version of the Rheumatoid Arthritis Stigma Scale (C-ISMI-RA)

This scale was revised by Geng et al. (2022). It uses a 4-point Likert scale, with a total score ranging from 20 to 80. A higher score indicates a more severe stigma. The Cronbach’s α coefficient for this scale is 0.90. The confirmatory factor analysis showed RMSEA = 0.09, CFI = 0.94, and TLI = 0.93.

### Health Assessment Questionnaire-Disability Index (HAQ-DI)

Consisting of 20 items, this scale is used to assess the physical function of RA patients. Scores are categorized as mild (total score ≤ 1), moderate (1 < total score ≤ 2), and severe (2 < total score ≤ 3). The scale exhibits good reliability and validity, making it suitable for evaluating physical function limitations in RA patients (Fries et al., 1980).

### Statistical analysis

Data analysis was conducted using SPSS 19.0 software. Descriptive statistical analysis was performed using means, standard deviations (SDs), medians, quartiles, absolute frequencies, and relative frequencies. Pearson’s correlation coefficient was used to explore the relationship between quantitative variables. Due to the inability to determine normal distribution previously, the Kruskal-Wallis test was used to determine the effect of variables on HL. Linear regression analysis was used to explore predictive associations among variables. A *p*-value of <0.05 indicates statistical significance.

## Results

### Common method deviation test

The Harman single-factor test was used to assess the common method deviation in the samples. The results indicate that the interpretation rate of the first factor is 15.5%, which is significantly below the critical threshold of 40% (Podsakoff et al., 2003), thus suggesting that there is no significant common methodological bias in the data from this sample.

### Patient characteristics

A total of 172 RA patients who met the inclusion criteria were surveyed, and 172 valid questionnaires were returned (response rate of 100%). The majority of RA patients were female, 72.67% were urban residents, and their ages ranged from 46 to 59 years, with a median age of 53 years. Most patients had a morning stiffness level of 1 (69.19%). Specific information on the patients is presented in Table 1.

**Table 1:** Characteristics of RA patients.

Variable	Mean ± SD or *N* (%)	Median (Q1,Q3)
Gender		
Male	22 (12.79)	
Female	150 (87.21)	
Age	52.62 ± 12.25	53.00 (46.00,59.00)
Illness duration	8.67 ± 8.06	5.65 (3.00,10.50)
District		
Urban	125 (72.67)	
Rural	47 (27.33)	
Education		
Primary school and below	51 (29.65)	
Junior school or technical school	78 (45.35)	
High school and above	43 (25.00)	
Occupation		
Employed	111 (64.53)	
Unemployed	61 (35.47)	
Income		
<3,000	88 (51.16)	
3,000–5,000	60 (34.88)	
>5,000	24 (13.95)	
Insurance		
Self-paid	11 (6.40)	
NCMS	37 (21.51)	
Basic medical insurance	124 (72.09)	
Family history		
Yes	17 (9.88)	
No	155 (90.12)	
Morning stiffness		
Level one	119 (69.19)	
Level two	35 (20.35)	
Level three	18 (10.47)	
Regular medication		
Level one	12 (6.98)	
Level two	45 (26.16)	
Level three	115 (66.86)	
ESR	33.44 ± 23.96	28.00 (16.50,45.00)
VAS	4.13 ± 2.09	4 (3,6)
PGA	3.89 ± 1.92	4 (3,5)
Joint pain	4.62 ± 5.02	3 (2,6)
joint swelling	3.09 ± 3.22	2 (1,5)
DAS28	4.48 ± 1.59	4.55 (3.06,5.62)

ESR, erythrocyte sedimentation rate; VAS, visual analog scale of pain; PGA, patient global assessment; DAS28, disease activity index.

### Scores on related scales for RA patients

The average scores for health literacy, stigma of illness, medication adherence, self-efficacy, and disability index were (83.54 ± 17.43), (54.51 ± 9.68), (70.16 ± 11.24), (84.91 ± 14.37), and (0.26 ± 0.44) respectively. Table 2 presents the detailed results.

**Table 2:** Descriptive statistics of related scales.

Variables	Mean (SD)	Median (Q1,Q3)
Health literacy (Health Literacy Scale for chronic patients)	83.54 (17.43)	81.00 (67.00,98.5)
Stigma (C-ISMI-RA)	54.51 (9.68)	55.00 (48.50,61.00)
Chinese Compliance Questionnaire for Rheumatology(CCQR)	70.16 (11.24)	66.67 (61.40,78.95)
Self-efficacy (C-RASE)	84.91 (14.37)	87.00 (76.00,94.50)
Health assessment Questionnaire-Disability index (HAQ-DI)	0.26 (0.44)	0.05 (0.0.35)

### The relationship between demographic, clinical variables, related scale scores, and HL

**Figure 1:**
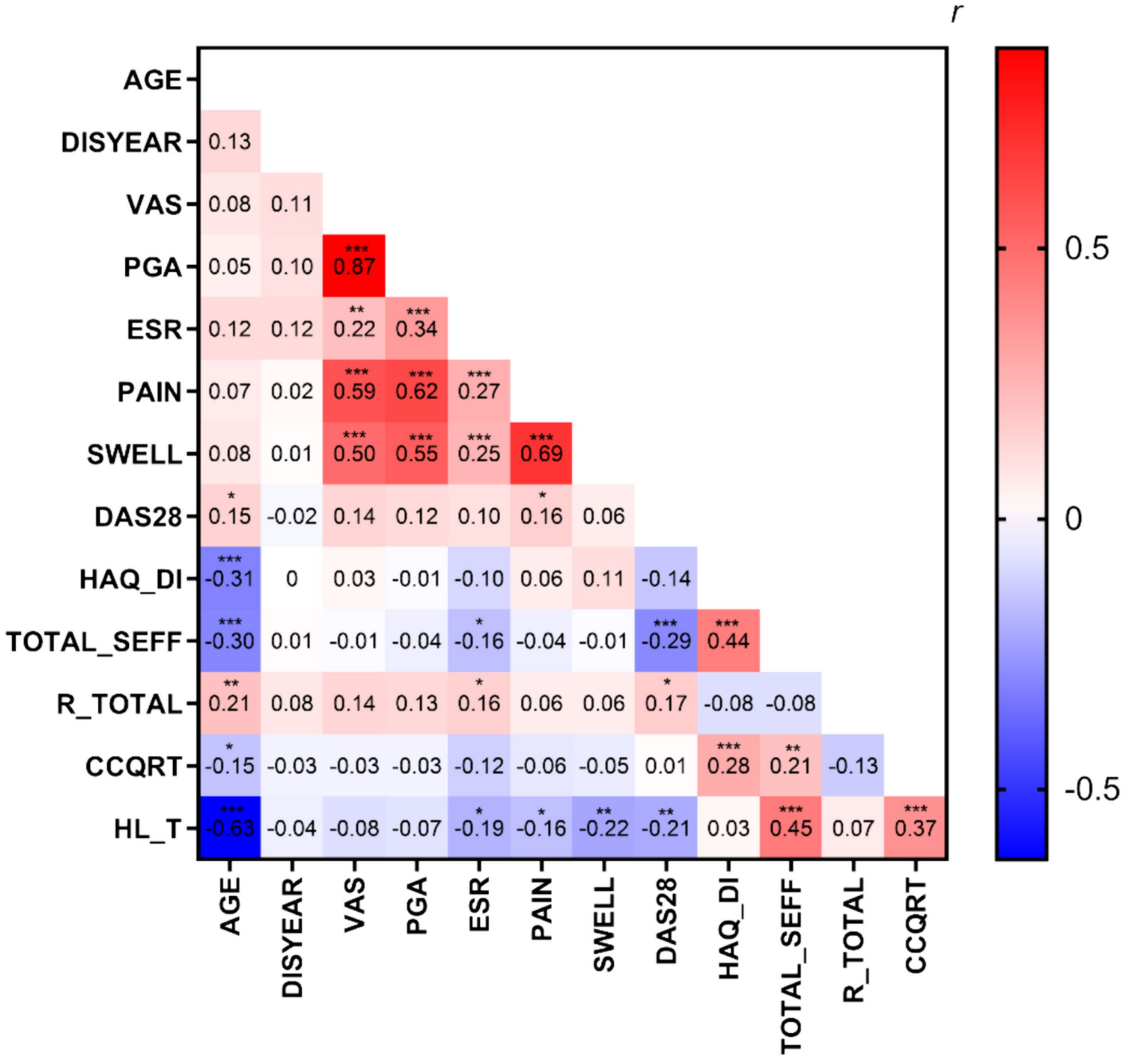
The heatmap of correlations between variables. DISYEAR, illness duration (month); ESR, erythrocyte sedimentation rate; pain, the total joint pain count; SWELL, the total joint swelling count; DAS28,disease activity index; HAQ-DI, total score of HAQ-DI; TOTAL_SEFF, total score of C-RASE; R_TOTAL, total score of C-ISMI-RA; CCQRT, total score of CCQR; HL_T, total score of HL Scale for chronic patients. *p < 0.05, **p < 0.01, ***p < 0.001.

As shown in Figure 1, The Spearman’s rank correlation coefficient was calculated to determine the relationship between HL (represented by HL_T) of RA patients and demographic and clinical variables. The results revealed that HL had a significant negative correlation with age (*r* = −0.63, *p* < 0.001), ESR (*r* = −0.19, *p* < 0.05), joint pain score (*r* = −0.16, *p* < 0.05), joint swelling score (*r* = −0.22, *p* < 0.01), and DAS28 (*r* = −0.21, *p* < 0.01), while it had a significant positive correlation with Total_SEFF (*r* = 0.45, *p* < 0.001) and CCQRT (*r* = 0.37, *p* < 0.001). The disease duration, pain score (VAS), PGA, and stigma score showed no significant correlation with HL and were not included in subsequent analyses.

### Univariate analysis of HL in RA patients

The results of the univariate analysis indicated that there were statistically significant differences in HL among patients with respect to age, disease activity (DAS28), self-efficacy, and treatment compliance (*p* < 0.05). Although HAQ did not have a significant correlation with HL (*F* = 1.44, *p* = 0.15), it was still included in the subsequent analysis for clinical consideration, as shown in Table 3.

**Table 3:** Univariable linear regression analysis between associated variables and HL.

Variables	*F*	*p*
Age	111.73	<0.001
DAS28	9.53	<0.001
TOTAL_SEFF	43.22	<0.001
CCQRT	28.79	<0.001

### Predictors of HL in RA patients

To determine which factors best predict HL, a stepwise multiple linear regression analysis was performed (Table 4). Spearman’s rank correlation coefficient was calculated to distinguish the relationships between these factors and HL (represented by HL_T score). Age, DAS28, HAQ_DI, self-efficacy, and CCQRT values were identified as predictors of HL. These predictors yielded a statistically significant model (*F* = 48.96, *p* ≤ 0.001), explaining 77% of the variation in the dependent variable (adjusted *R*^2^ = 0.765). The final equation is: HL = 64.67–0.82*Age - 4.20*DAS28 + 12.29*HAQ_DI + 0.46*TOTAL_SEFF +0.51*CCQRT.

**Table 4:** Multiple linear regression analysis explaining the variance of HL in RA patients (*n* = 172).

Variable	*β*	SE	Beta	*t*	*p*	*R*^2^	Adjusted *R*^2^	*F*
Intercept	64.67	7.63	0.00	8.47	<0.001	0.774	0.765	48.96
Age	−0.82	0.05	−0.58	−15.34	<0.001			
DAS28	−4.20	0.53	−0.33	−7.99	<0.001			
HAQ_DI	12.29	1.66	0.31	7.39	<0.001			
TOTAL_SEFF	0.46	0.05	0.38	10.26	<0.001			
CCQRT	0.51	0.06	0.33	8.86	<0.001			

DAS28, disease activity index; HAQ-DI, total score of HAQ-DI; TOTAL_SEFF, total score of C-RASE; R_TOTAL, total score of C-ISMI-RA; CCQRT, total score of CCQR; HL_T, total score of Health Literacy Scale for chronic patients.

## Discussion

The average HL score for RA patients in China is (83.54 ± 17.43), indicating a moderate level. Although it is still lower than the diabetic patients (Qin and Xu, 2016), significant progress has been made in the “Healthy China 2030” strategy. An effective way to improve HL among RA patients is to implement chronic disease management. China started relatively late in this regard, but China’s still actively exploring a chronic rheumatic disease management model that suits its national conditions. HL emphasizes active acquisition, critical thinking, and ultimately using the obtained information to make healthy decisions and solve problems. Foreign studies (Hirsh, 2016) have shown that low education levels can affect HL levels. In this study, 75% of the participants had an education level below high school, which created certain obstacles in accessing and utilizing health information, and may be a reason why the HL score of RA patients is at a moderate level.

Self-efficacy refers to individuals’ confidence or belief in their ability to achieve specific behavioral goals in a particular domain (Cepukiene and Puzeriene, 2024). The average score of self-efficacy for RA patients in this study was (84.91 ± 14.37), indicating that it is consistent with a cross-sectional study in Spain (Cantero-Tellez et al., 2023). The results of the multivariate analysis show that self-efficacy is a predictor of HL among RA patients. RA patients with higher HL levels are more likely to be aware of the relationship between current behaviors and health outcomes, which plays an important role in adopting and maintaining healthy behaviors (Liu et al., 2024). The study by Cantero-Tellez et al. has proven that individuals with high self-efficacy are more likely to adopt positive health behaviors (Cantero-Tellez et al., 2023). HL, self-efficacy, and their levels are closely related to health outcomes and quality of life. Enhancing self-efficacy and improving HL among RA patients, as well as changing unhealthy lifestyles, can contribute significantly to improving their quality of life.

The proportion of medication adherence among RA patients ranges from 30 to 93% (Omair et al., 2022). Some studies have shown that low HL in RA patients is associated with poor medication adherence (Contreras-Yanez et al., 2021). In this study, there was a positive correlation between self-efficacy and medication adherence (*p* < 0.01), which is consistent with the findings of Lavsa’s research (Lavsa et al., 2011). Simultaneously, there was also a positive correlation between disability index and medication adherence (*p* < 0.001), in line with Nagafusa’s study (Nagafusa et al., 2023). Multivariate analysis revealed that medication adherence is a predictor of HL. Gomez-Galicia and colleagues reported that low HL is associated with poor self-reported medication adherence among RA patients (Gomez-Galicia et al., 2022). Hirsh et al. (2020) pointed out that patients with low HL encounter difficulties in reading medication labels and prescriptions. Additional research has shown that patients with low levels of light physical activity and self-efficacy tend to have severe joint deformities and more functional limitations (Contreras-Yanez et al., 2021 Park et al., 2024). In this study, medication adherence and self-efficacy both affect HL among RA patients, but we do not know whether these two factors directly impact HL or if one factor indirectly affects HL through the other. This remains to be further explored in the future. However, it is certain that interventions targeting HL can improve patients’ long-term medication adherence, increase treatment engagement, and enhance skills in medication management.

In this study, the mean value of the Health Assessment Questionnaire Disability Index (HAQ-DI) was 0.26 ± 0.44, indicating mild functional disability. Univariate regression analysis showed no significant correlation between HAQ-DI and HL. However, based on clinical judgment, HAQ-DI was included in the multiple regression analysis, suggesting its potential as a predictor of HL. This may be related to the data sources, since most of the data came from chronic rheumatic disease management centers. Domestic experts believe that to address physical dysfunction among RA patients in China, more attention should be paid to elderly patients and those with high disease activity (Zhou et al., 2018). Currently, there are few studies on the relationship between physical dysfunction and HL, but some research has shown a close relationship between low HL and cognitive impairment (Han et al., 2015). Therefore, we hypothesize that the disability index may predict HL among RA patients, and that there may be an underlying relationship with cognitive impairment, which requires further validation.

Age (52.62 ± 12.25) and the average DAS28 score (4.48 ± 1.59) were also significant negative factors for HL. Among the recruited age range, older age was associated with lower HL. These findings are similar to previous studies (Buchbinder et al., 2006), which suggest that reading, writing, and cognitive abilities decline as patients age (Fraenkel et al., 2006). HL emphasizes the ability to read, write, access information, communicate, and understand. In terms of disease activity, higher DAS28 scores were associated with lower HL, possibly because patients experience recurrent disease episodes and poor control, leading to a complete loss of confidence in self-management. They may perceive the disease as an incurable cancer and shut themselves off from all disease-related information. Therefore, controlling the disease in a stable state is of utmost importance to improve HL.

## Conclusion

This study shows that in Chinese RA patients, HL is negatively correlated with age, ESR, the number of tender joints, the number of swollen joints, and disease activity score (DAS28), while positively correlated with self-efficacy and medication adherence. A preliminary prediction model for HL has been established, providing a theoretical basis for healthcare professionals to develop targeted strategies to effectively enhance functional, critical, and interactive HL levels, strengthen patients’ ability to access self-disease management information, and improve their quality of life (Nutbeam and Lloyd, 2021). However, there are some limitations to this study. Although our research initially established a prediction model for HL in RA patients, covering a broad range, we still need to expand the sample size and conduct a multi-center study to validate the model in the future. Moreover, a longer follow-up time would be beneficial to further validate the model.

## Data availability statement

The original contributions presented in the study are included in the article/supplementary material, further inquiries can be directed to the corresponding author.

## Ethics statement

The studies involving humans were approved by Ethics Committee of Changzhou Second People’s Hospital. The studies were conducted in accordance with the local legislation and institutional requirements. The participants provided their written informed consent to participate in this study.

## Author contributions

TL: Writing – original draft, Writing – review & editing, Data curation. WM: Conceptualization, Data curation, Writing – original draft, Writing – review & editing. WW: Formal analysis, Project administration, Writing – original draft, Writing – review & editing. GS: Data curation, Investigation, Methodology, Resources, Writing – original draft, Writing – review & editing. XC: Data curation, Investigation, Resources, Writing – original draft, Writing – review & editing. YL: Supervision, Visualization, Writing – original draft, Writing – review & editing. WQ: Writing – original draft, Writing – review & editing, Investigation. YW: Data curation, Resources, Supervision, Writing – original draft, Writing – review & editing. LZ: Formal analysis, Investigation, Writing – original draft, Writing – review & editing. SZ: Supervision, Validation, Writing – review & editing, Writing – original draft.
